# The effect of parental age on the presence of de novo mutations – Lessons from neurofibromatosis type I

**DOI:** 10.1002/mgg3.222

**Published:** 2016-06-16

**Authors:** Tom Dubov, Hagit Toledano‐Alhadef, Felix Bokstein, Shlomi Constantini, Shay Ben‐Shachar

**Affiliations:** ^1^ Sackler Faculty of MedicineTel‐Aviv UniversityTel‐AvivIsrael; ^2^ The Gilbert Israeli Neurofibromatosis CenterTel‐Aviv Medical CenterTel‐AvivIsrael; ^3^ Department of Pediatric NeurosurgeryDana Children's Hospital, Tel‐Aviv Medical CenterTel‐AvivIsrael

**Keywords:** Advanced paternal age, autosomal dominant, de novo, sporadic neurofibromatosis type 1

## Abstract

**Background:**

Neurofibromatosis type 1 (NF1) is the most common autosomal dominant neurocutaneous disease with a prevalence of 1:2500. Approximately, 50% of the cases are sporadic. Advanced paternal age is associated with germline mutations and autosomal diseases. We aimed to use NF1 as a paradigm to study the effect of parental age on sporadic mutation rates for both advanced and younger parental ages.

**Methods:**

The medical charts of 118 NF1 pediatric patients followed in a specialized Israeli NF1 clinic were evaluated. Thirty‐one cases were diagnosed by genetic tests and 87 by NIH clinical criteria. Sixty‐four cases (54%) had a negative family history of NF1 (sporadic cases). Data on parental ages at the time of the children's birth were compared to the national population database.

**Results:**

Parental age of children with sporadic NF1 was higher than the general population (32.7 years vs. 30.1 years, respectively, for the mothers and 36.5 years vs. 32.6 years, respectively, for the fathers; *P* < 0.0001 for both groups). In contrast, the age of the mothers and the fathers in the familial cases (30.3 and 33.9 years, respectively) did not differ from the general population. Significantly, fewer fathers of the sporadic group had been 25–29 years old at their child's birth compared with fathers in the general population (7.8% vs. 21%, respectively, *P* = 0.009), and significantly more fathers were ≥40 years old (29.7% vs. 13.6%, respectively, *P* = 0.0002). Differences in maternal age between these two groups were less prominent.

**Conclusion:**

Parents of sporadic NF1 cases are older. The risk for sporadic NF1 was lower when the fathers were younger at the time of the affected child's birth, and gradually increased with paternal age.

## Introduction

While advanced maternal age (AMA) is associated with aneuploidy (Hook and Chambers 1977; Hook and Lindsjo 1978; Warren et al. 1987), advanced paternal age (APA) is associated with sporadic occurrence of a number of autosomal dominant (AD) genetic disorders such as achondroplasia (Penrose 1957; Thompson et al. 1986), Marfan syndrome (Murdoch et al. 1972), and osteogenesis imperfecta (Carothers et al. 1986). It has been shown that the mutations in those disorders occurred mainly on the paternal inherited allele, suggesting that they occur preferentially during spermatogenesis (Wilkin et al. 1998; Goriely and Wilkie 2010, 2012; Yoon et al. 2013). Interestingly, APA is associated not only with pure Mendelian disorders but with complex disorders as well, such as autism (Durkin et al. 2008; Hultman et al. 2011) and schizophrenia (Kuhnert and Nieschlag 2004). These sporadic cases usually represent an occurrence of de novo mutation in the affected proband. However, they may rarely represent the existence of a parental germline/somatic mosaicism.

A number of mechanisms were proposed to cause an increased mutation rate during the spermatogenesis of older males including replication errors and reduced activity of repair enzymes (Crow 2000). In addition, the human sperm DNA is more methylated than oocyte DNA, which may account for the greater number of point mutations occurring within a CpG dinucleotide (Glaser and Jabs 2004). Another mechanism for the paternal age effect was suggested in recent studies based on direct quantification of mutations in sperms and testes (Goriely et al. 2003). The paternal age effect was shown to lie in the dysregulation of spermatogonial cell behavior mediated through the growth factor receptor‐RAS signal transduction pathway. This theory suggests that the mutations are positively selected and that they expand clonally in normal testes, leading to enrichment of mutant sperm over time (Goriely and Wilkie 2010, 2012).

Friedman calculated the risk for de novo AD mutations to be 0.3–0.5% among the offspring of fathers aged >40 years. This risk was similar in magnitude to the risk of Down syndrome among the offspring of 35‐ to 40‐year‐old mothers (Friedman 1981). The cutoff for defining advanced paternal age was set at >40 years (Friedman 1981; de La Rochebrochard and Thonneau 2003; Toriello and Meck 2008). However, the cutoff at which APA begins to harbor a risk for de novo mutations and whether a young paternal age is protective against sporadic NF1 have not been conclusively determined.

Neurofibromatosis type 1 (NF1, MIM #162200) is a common AD neurocutaneous disease, caused by mutations in the *NF1* tumor suppressor gene (MIM # *613113), with a prevalence of 1:2000–1:5000 (Rasmussen and Friedman 2000; Evans et al. 2010). The *NF1* gene encodes the protein neurofibromin, which is a negative regulator of the Ras oncogene and its downstream effectors, including Ras/mitogen‐activated protein kinase (MAPK). Similar to other genetic disorders related to the Ras‐MAPK pathway, NF1, belongs to a group of genetic disorders termed “Rasopathy” (Martin et al. 1990; Upadhyaya et al. 2007; Gottfried et al. 2010).

While its clinical signs appear gradually, the disease eventually has a complete penetrance, enabling easy clinical recognition of sporadic cases, which account for about 50% of the patients (Sergeyev 1975; Riccardi et al. 1984; Samuelsson and Akesson 1989; Clementi et al. 1990; Takano et al. 1992; Poyhonen et al. 2000; Snajderova et al. 2012). The reported parental origin of the majority of de novo *NF1* mutations is the paternal germline (Jadayel et al. 1990; Stephens et al. 1992). Most of the studies that explored the association between APA and NF1 reported a paternal age in the sporadic NF1 group that was higher by 1.5–3.5 years comparedwith the general population. The majority of those studies did not find significant difference in the mean maternal age (Sergeyev 1975; Riccardi et al. 1984; Samuelsson and Akesson 1989; Jadayel et al. 1990; Takano et al. 1992; Poyhonen et al. 2000; Snajderova et al. 2012; Liu et al. 2015). However, some studies failed to find any parental age differences (Huson et al. 1989; Jadayel et al. 1990).

Given the clear NF1 phenotype, its high prevalence, the high rate of de novo mutations, and the possibility that mutations in genes related to the RAS‐MAPK pathway might be associated with an increased de novo mutation rate (Yoon et al. 2013), we aimed to use NF1 as a paradigm. We strived to investigate the effect of paternal age on the rate of de novo mutations, not only for advanced paternal ages but for younger ages as well.

## Materials and Methods

### NF1 patients

The medical charts of the patients aged 0–17 years of Jewish ethnicity, who were referred to genetic evaluation in a tertiary neurofibromatosis center, during 2004–2014, were reviewed. NF1 was diagnosed based either on the National Institutes of Health (NIH) clinical criteria (DeBella et al. 2000; Ferner et al. 2007) or on the existence of a single NIH clinical criteria and a disease‐causing mutation detected in blood leukocytes. Sporadic cases were determined according to the detailed pedigree information and the absence of parental NF1 signs. Familial cases were defined when one of the affected child's parents met the NIH clinical criteria for NF1.

### Israeli population

Data on both maternal and paternal ages of the cohort were compared to data obtained from the national population database at the Ministry of Health, which is updated monthly for the Israeli population by the Ministry of Internal Affairs.

### Ethical compliance

The study was approved by the institutional ethics review board of the Tel‐Aviv Medical Center.

### Statistical analysis

The *t*‐test was used to compare the parental ages of the sporadic and familial NF1 groups and the general population. The Pearson chi‐square test was used to examine a possible relationship between parental age groups and NF1 prevalence. The Sidak method for adjustment of significance level was employed (Winer et al. 1991). A Z‐test for two population proportions was implemented when the results were significant. The significance level was adjusted by using the multiple comparisons adjustment method (Winer et al. 1991). Statistical analysis was performed by SAS (Cary, NC) version 9.2.

## Results

A total of 118 children were identified as having NF1, of whom 33.9% (40/118) were familial cases and 66.1% (78/118) were sporadic cases. Forty percent of the familial cases (16/40) and 51% (40/78) of the sporadic cases were males. Detailed information on parental age and family history was available for 64 (82%) of the sporadic cases and 31 of the familial cases (78%). The mean age of the participants was 6.3 ± 4.8 years at initial visit for the sporadic cases and 7.7 ± 4.2 years for the familial cases. The mean paternal and maternal ages were 36.5 ± 6.8 and 32.7 ± 5.4 years, respectively for the sporadic cases and 33.9 ± 5.3 and 30.3 ± 5.5 years, respectively, for the familial cases. There was an overall trend toward higher parental age for the sporadic cases (*P* = 0.056 for parental age and *P* = 0.039 for maternal age.

Given that the median age of the sporadic NF1 group was 6.3 ± 4.8 years, the parental age was compared to the parental age of the Jewish Israeli population of children born 6 years prior to the acquisition of data (2009, Tables 1 and 2).

**Table 1:** Comparison between paternal age of sporadic neurofibromatosis type 1 cases and Israeli population by age groups

Age, years	Israeli population	NF1	*P*‐value
<25			
Frequency	17,068	2	0.171
Percent	7.70	3.13	
25 ≤ age < 30			
Frequency	46,611	5	0.009^a^
Percent	21.03	7.81	
30 ≤ age < 35			
Frequency	73,058	15	0.105
Percent	32.97	23.44	
35 ≤ age < 40			
Frequency	54,730	23	0.038
Percent	24.70	35.94	
40≤			
Frequency	30,124	19	<0.001^a^
Percent	13.59	29.69	
Total	221,591	64	

a Significant after statistical adjustment for multivariable test.

**Table 2:** Comparison between maternal age of sporadic neurofibromatosis type 1 cases and Israeli population by age groups

Age, y	Israeli population	NF1	*P*‐value
<25			
Frequency	33,527	6	0.25
Percent	14.41	9.38	
25 ≤ age < 30			
Frequency	66,278	8	0.005^a^
Percent	28.49	12.50	
30 ≤ age < 35			
Frequency	76,602	27	0.114
Percent	32.93	42.19	
35 ≤ age < 40			
Frequency	44,258	17	0.123
Percent	19.03	26.56	
40≤			
Frequency	11,949	6	0.123
Percent	5.14	9.38	
Total	232,614	64	

a Significant after statistical adjustment for multivariable test.

**Figure 1:**
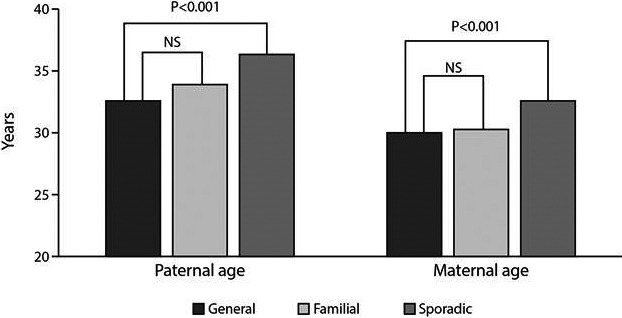
Mean maternal and paternal age at child birth among the general Israeli population during 2009, familial NF1cases, and sporadic NF1 cases.

The average parental age at birth for the general Jewish population was 32.6 ± 6.1 years for paternal age and 30.1 ± 5.4 for maternal age. These ages were similar to the parental age of the familial cases (32.7 ± 5.4 and 30.3 ± 5.5 for paternal and maternal ages, respectively). In contrast, the parental ages of the sporadic group were older compared with the general population, by 3.9 years for the fathers and 2.6 years for the mothers (*P* < 0.0001 for both parental and maternal ages, Fig. 1).

In order to detect the specific ages that had contributed to these differences in parental ages, the parental ages were divided into age bins of 5 years each.

**Figure 2:**
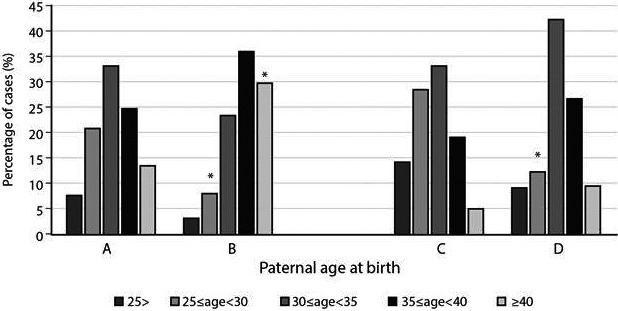
Parental age distribution of the general Israeli population and sporadic neurofibromatosis type I (NF1) cases. Percentage of fathers in the different age groups at the time of the child's birth (A) as derived from the national data and (B) in the sporadic NF1 study population. Percentage of mothers in the different age groups at the time of the child's birth (C) as derived from the national data and (D) in the sporadic NF1 study population. *Represents statistical significance (*P* < 0.05) after correction for multiple testing.

It emerged that 7.7% of the fathers in the general Israeli Jewish population were younger than 25 years compared with only two fathers (3.13%) of the sporadic NF1 group. This difference was, however, not significant. Accordingly, 21% (46,611/221,591) of the Israeli fathers in the general population and 7.8% (5/64) of fathers in the sporadic NF1 group were 25–29 years old (*P* = 0.0093). In contrast, 13.6% (30,124/221,591) of the Israeli fathers, and 29.7% (19/64) of the fathers of the sporadic NF1 group were older than 40 years at the time of birth (*P* = 0.00016). A similar trend was shown for fathers in the 35–39 years age group, but it did not remain significant after statistical adjustment (Table 1, Fig. 2A,B).

Assessment of the maternal group revealed that 28.5% (66,278/232,614) of the mothers in the Israeli population and 12.5% (8/64) of mothers in the sporadic NF1 group were 25–29 years old at the time of their child's birth (*P* = 0.005). No other significant differences were detected for the other age groups (Table 2, Fig. 2C,D). Given the small size of the Jewish familial NF1 cohort (31 patients), we could not divide that group into subgroups based on ages.

## Discussion

While maternal age is a major risk factor for fetal aneuploidy, the age‐related risk factor is only mildly increased among younger women and increases significantly with more advanced maternal ages (Hook and Chambers 1977; Hook and Lindsjo 1978). Similarly, the association between APA and de novo mutations occurring in sperm is well established (Penrose 1957; Murdoch et al. 1972; Carothers et al. 1986; Thompson et al. 1986; Glaser et al. 2000). These mutations may be positively selected and they may expand clonally in normal testes, leading to enrichment of mutant sperm over time (Goriely et al. 2003, 2005; Goriely and Wilkie 2010, 2012; Yoon et al. 2013). Moreover, based on this paradigm, de novo events have been suggested to contribute to an array of complex disorders, such as autism, in which an association between APA and increased disease incidence was found (Durkin et al. 2008; Hultman et al. 2011). However, the precise characteristics of the paternal age effect have not been fully identified. Such information is essential for both understanding the biological basis of this effect as for practical genetic counseling purposes. For example, the increased rate of autosomal trisomies in older females has led to a worldwide recommendation for performing invasive prenatal testing for women above the age of 35 years.

This study provides information on the association between parental age and the occurrence of sporadic NF1. The results show not only the effect of APA, but that the affected individuals have under‐representation of younger fathers (25–30 years). A similar pattern was observed for fathers younger than 25 years, but it did not reach a level of significance, possibly due to the relatively small numbers of fathers of affected children in that group. A similar pattern was revealed for maternal age. However, this pattern was less prominent and reached significance solely for the 25‐ to 30‐year age group.

The overall age pattern for both parental sexes was skewed from the normal bell‐shaped curve seen in the general population for both younger and older parental ages (Fig. 2). It was, however, more prominent for the paternal age. This observed difference is in agreement with the findings in the majority of previous studies (Sergeyev 1975; Riccardi et al. 1984; Samuelsson and Akesson 1989; Takano et al. 1992; North 1993; Bunin et al. 1997; Snajderova et al. 2012; Liu et al. 2015). A few studies did not find this effect, perhaps due to the small number of subjects (Huson et al. 1989; Jadayel et al. 1990). Since most of the sporadic *NF1* mutations occurred in the paternal germline (Jadayel et al. 1990; Stephens et al. 1992), it is possible that the detected maternal age effect represents a secondary effect.

This study, uniquely, demonstrated that there is, indeed, a paternal age effect that related not only to APA but to different age groups as well. It appears that the risk is lower when the fathers are younger and that it gradually increases with paternal age. Since there are multiple de novo autosomal dominant disorders associated with APA, this finding may hint to an overall biological advantage of young fatherhood.

An APA effect associated with de novo mutations causing AD disorders has been detected for most of the tested disorders (Risch et al. 1987). It is, therefore, possible that the pattern observed for NF1 in this study may be applied to other conditions associated with de novo mutations, and that early parenthood for males may have a significant impact on their children's health. Moreover, it is possible that the younger paternal age may also have some protective effect against common complex disorders, such as autism. These possibilities warrant further in‐depth studies since the consequences may be far‐reaching if evidence‐based conclusions can be provided on these important issues.

This study has some limitations. First, it is retrospective in design, which may affect the quality of the data. Second, the possibility of germline mosaicism and non‐paternity cannot be excluded in all apparently sporadic cases. However, any existing germline mutations in the parents may decrease the observed parental effect and, therefore, are not expect to affect the observed results.

## Conflict of Interest

The authors declare that they have no conflict of interest.
